# MF-MNER: Multi-models Fusion for MNER in Chinese Clinical Electronic Medical Records

**DOI:** 10.1007/s12539-024-00624-z

**Published:** 2024-04-05

**Authors:** Haoze Du, Jiahao Xu, Zhiyong Du, Lihui Chen, Shaohui Ma, Dongqing Wei, Xianfang Wang

**Affiliations:** 1 Department of Computer Science, North Carolina State University, Raleigh, NC 27695 USA; 2 School of Computer Science and Technology, Henan Institute of Technology, Xinxiang, 453003 China; 3 School of Electrical and Electronic Engineering, Nanyang Technological University, Singapore, 639798 Singapore; 4 State Key Laboratory of Microbial Metabolism, School of Life Sciences and Biotechnology, Shanghai Jiaotong University, Shanghai, 200240 China; 5 Joint Laboratory of International Cooperation in Metabolic and Developmental Sciences, Ministry of Education, Shanghai Jiaotong University, Shanghai, 200240 China; 6 Zhongjing Research and Industrialization, Institute of Chinese Medicine, Zhongguancun Scientific Park, Nanyang, 473000 China

**Keywords:** Multi-models fusion, MNER, BART, Bi-LSTM, CRF

## Abstract

To address the problem of poor entity recognition performance caused by the lack of Chinese annotation in clinical electronic medical records, this paper proposes a multi-medical entity recognition method F-MNER using a fusion technique combining BART, Bi-LSTM, and CRF. First, after cleaning, encoding, and segmenting the electronic medical records, the obtained semantic representations are dynamically fused using a bidirectional autoregressive transformer (BART) model. Then, sequential information is captured using a bidirectional long short-term memory (Bi-LSTM) network. Finally, the conditional random field (CRF) is used to decode and output multi-task entity recognition. Experiments are performed on the CCKS2019 dataset, with *micro avg Precision*, *macro avg Recall*, *weighted avg Precision* reaching 0.880, 0.887, and 0.883, and *micro avg F1-score*, *macro avg F1-score*, *weighted avg F1-score* reaching 0.875, 0.876, and 0.876 respectively. Compared with existing models, our method outperforms the existing literature in three evaluation metrics (*micro average*, *macro average*, *weighted average*) under the same dataset conditions. In the case of weighted average, the *Precision*, *Recall*, and *F1-score* are 19.64%, 15.67%, and 17.58% higher than the existing BERT-BiLSTM-CRF model respectively. Experiments are performed on the actual clinical dataset with our MF-MNER, the *Precision*, *Recall*, and *F1-score* are 0.638, 0.825, and 0.719 under the micro-avg evaluation mechanism. The *Precision*, *Recall*, and *F1-score* are 0.685, 0.800, and 0.733 under the macro-avg evaluation mechanism. The *Precision*, *Recall*, and *F1-score* are 0.647, 0.825, and 0.722 under the *weighted avg* evaluation mechanism. The above results show that our method MF-MNER can integrate the advantages of BART, Bi-LSTM, and CRF layers, significantly improving the performance of downstream named entity recognition tasks with a small amount of annotation, and achieving excellent performance in terms of recall score, which has certain practical significance. Source code and datasets to reproduce the results in this paper are available at https://github.com/xfwang1969/MF-MNER.

**Graphical Abstract:**

Illustration of the proposed MF-MNER. The method mainly includes four steps: (1) medical electronic medical records need to be cleared, coded, and segmented. (2) The semantic representation obtained by dynamic fusion of the bidirectional autoregressive converter (BART) model. (3) The sequence information is captured by a bi-directional short-term memory (Bi-LSTM) network. (4) the multi-task entity recognition is decoded and output by conditional random field (CRF). 
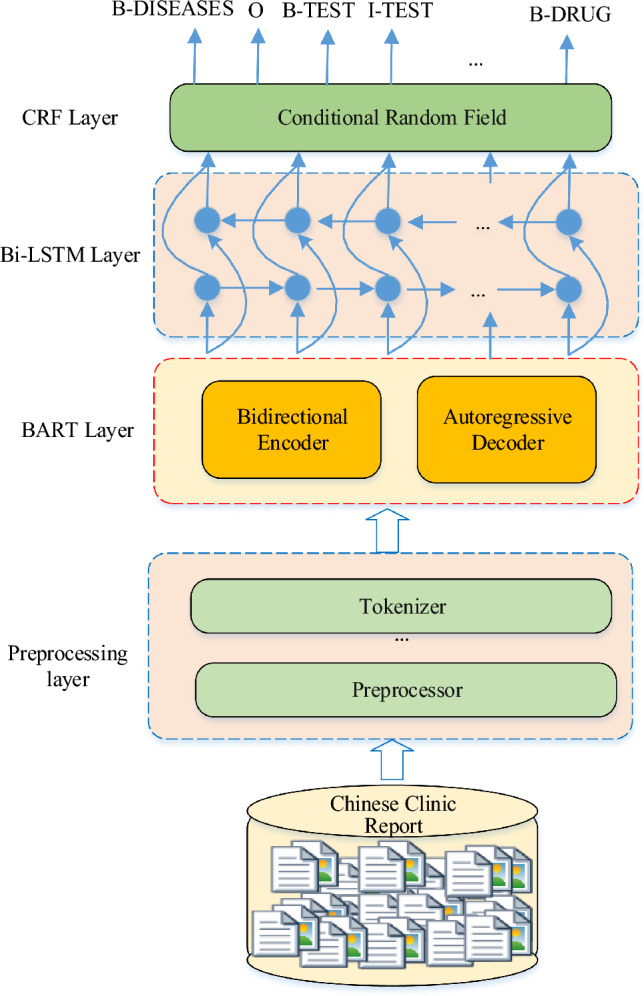

## Introduction

With the development of electronic medical information, numerous hospitals have generated a vast amount of clinical electronic medical. These electronic medical records, as a form of unstructured data, often contain key diagnostic information such as patients' clinical symptoms, diagnostic results, and medication performance [1, 2]. They are rich with medical knowledge, and the accurate and rapid extraction of relevant medical named entity information from them is fundamental to disease research. This plays a crucial role in clinical decision support, medical information retrieval, Medical intelligent question and answer, medical entity relationship information retrieval, and more [3–6]. Medical Named Entity Recognition (MNER) is the basis for medical information relationship recognition, is crucial to smart medical care, and has received widespread attention from researchers [7, 8]. However, clinical medical data has the problem of being difficult to accurately identify due to the small amount of annotated data. How to use a small amount of annotated data to establish an accurate clinical named entity recognition model is one of the key tasks in medical information processing [1, 4].

The earliest research focused on named entity recognition for English electronic medical records in the medical field [9, 10], followed by some researchers who designed methods for recognizing Chinese electronic medical records [11, 12]. From a technical perspective, both named entity recognition in Chinese electronic medical records and English electronic medical records can be roughly divided into four types: dictionary and rule-based methods, statistical learning methods, neural network learning methods, and pre-training methods based on large-scale unlabeled data. Dictionary and rule-based methods mainly rely on a large and comprehensive domain dictionary and domain experts to construct many rule templates based on grammatical structures. However, this method cannot handle the recognition of new entities and irregular entities well and cannot be reused across different domains [13]. Feature engineering is a crucial part of statistical learning methods. It improves the performance of machine learning algorithms by preprocessing and extracting features from raw data to obtain a more effective and reliable feature set. Research using Conditional Random Fields [14], Support Vector Machines (SVM) [15], Hidden Markov Models (HMM) [16], etc., have achieved good results. However, these methods rely on feature sets created by high-quality feature engineering, The completion of this task relies heavily on manual efforts. The subjectivity and labor costs are relatively high, and the quality of feature selection directly affects the results of MNER. To address these issues, some scholars have proposed methods based on neural network learning. The most representative is the CNN-CRF model [17, 18] and the BiLSTM-CRF model [12, 19], which use neural networks and deep learning neural networks to autonomously pull out features at the character, word, and sentence tiers, lessening the subjectivity in feature selection and consequently enhancing the accuracy of recognition results [19]. Nonetheless, this technique demands high-quality annotated data in the medical field to ascertain the model's identification efficacy. The labeling data of medical clinical cases are often particularly limited. Obtaining these labeled data usually requires the participation of business personnel or even medical experts, which is especially costly and time-consuming [20]. Therefore, this series of methods is difficult to function as expected.

In recent years, pre-training methods utilizing massive-scale unlabeled data have provided new solutions [21]. Pre-trained language models (PLMs) trained on large-scale corpora, like BERT (Bidirectional Encoder Representations from Transformers), don't merely contain prior information from the training corpus in their dynamic word vectors but also include context information after the sentences are encoded by BERT [22]. Some researchers use LSTM-CRF (Long Short-Term Memory with Conditional Random Field) as the main framework to address the shortcomings of single neural network named entity recognition models [23]. To further enhance the model's ability to capture details and extract features, some studies have integrated Convolutional Neural Networks (CNN) and attention mechanisms into the BiLSTM-CRF model [19]. This has improved the recognition accuracy and injected new vitality into the field of MNER in English electronic medical records. However, it's worth mentioning that despite these advancements, there are still challenges to be addressed. The complexity of medical terminology and the variability in the way information is recorded in medical records can make named entity recognition more difficult [24, 25].

Gong L et al. established a BiLSTM-Att-CRF model to identify medical entities in the CCKS2017 dataset [26]. They only studied four entities (disease, symptom, drug, and surgery), and the average *Precision*, *Recall*, and *F*1-*score* only reached 75.06%, 76.40%, and 75.72%, respectively. Luqi Li et al. established a BiLSTM-Att-CRF model to identify medical entities in the CCKS2017 dataset and CCKS 2018 dataset [27]. Although the models achieved good performance. Due to the limited number of entities, it is not conducive to subsequent research on entity relationship extraction and knowledge graph construction. The Embeddings from Language Models (ELMo)-lattice-LSTM-CRF model was designed in literature [28] and achieved an F1 score of 85.02% on the CCKS-2019 CNER dataset. However, the performance indicators need to be further improved. Moreover, the privacy and sensitivity of medical data add another layer of complexity to the task. Therefore, while the integration of advanced techniques like pre-trained language models and attention mechanisms has greatly improved performance, there's still room for further research and development in this field.

To deal with the above challenges, this paper designs a named entity recognition method for Chinese electronic medical records named MF-MNER, which is mainly based on multi-model fusion and multi-task learning. Firstly, the original Chinese electronic medical record data is preprocessed, including sentence segmentation, word segmentation, named entity annotation, and construction of tag sequences, and the training set and validation set are obtained by randomly dividing the annotated data. Then, the BART (Bidirectional and Auto-Regressive Transformer) layer is used to convert the input sentences into fixed length and vectorize the input sequence. On this basis, a Bi-directional Long Short-Term Memory (Bi-LSTM) layer is constructed to process the input sequence from one end to the other and vice versa simultaneously, capturing the context information in the sequence. The final output layer uses a Conditional Random Field (CRF) layer, which classifies each position based on the features extracted by Bi-LSTM, outputs the probability distribution of each annotated sequence, and determines the output entities and their corresponding positions based on probability. The AdamW optimization algorithm is employed throughout the model's training phase for hyperparameter tuning, enabling the model to better predict named entity tags. This design approach is then assessed in the CCKS 2019 data set and in the real data set of named entity recognition in Chinese electronic medical records, proving its efficacy.

The remaining sections of this paper are structured as follows: Sect. 2 mainly introduces the work related to this research, including task definition, description of the meaning of the six medical entities identified, as well as the design of the multi-model fusion method and the key technologies involved. Section 3 mainly introduces the specific practical implementation process of each link of the model. The results from the experiments and their comparative analysis are discussed in Sect. 4. The paper is summarized and concluded in Sect. 5.

## Related Work

The general clinical medical electronic medical record naming entity mainly involves three stages: preprocessing, feature extraction, and model training. In English electronic medical records, words are clearly separated by spaces, making word segmentation relatively simple [20]. However, in Chinese electronic medical records, there is no obvious word separation, making word segmentation an important and complex step. In addition, preprocessing steps like purifying text and deleting stop words are also required [29]. In the feature extraction stage, in addition to common features such as word frequency and context information, Chinese electronic medical records may also need to consider specific features, such as the structural information of words (e.g., whether they are phrases or compound words). In the model training stage, Chinese named entity recognition can also use methods such as CRF, SVM, and deep learning, but the models need to be adjusted to adapt to the characteristics of Chinese [15]. This section mainly defines and formalizes the task of MNER and introduces the relevant technologies used in our research framework.

### Task Definition and Description

The task of MNER is to recognize and extract mentions of clinical medical entities from a provided collection of electronic health record texts and categorize them into predefined classes. Generally, Chinese MNER is a sequence tagging issue, including categories such as diseases & diagnoses, imaging examinations, lab tests, surgeries, medications, and anatomical sites [29]. The formal definition of MNER task can be defined as Eq. (1):

1 $$Y = G_M (D,C)$$

where $G_M$ is the model function, *D* is the input dataset, which consists of *N* electronic medical records $d_i$, represented as a set $C$ is the set of predefined categories {*c*_1_, *c*_2_, …, *c*_m_}, *Y* is the output of the model, which represents the set of named entity mentions and their corresponding categories. The objective of this research can be formulated as follows:

2 $${\text{Y}} = \left\{ {\left\langle {m_1 ,c_{m_1 } } \right\rangle ,\left\langle {m_2 ,c_{m_2 } } \right\rangle , \ldots ,\left\langle {m_p ,c_{m_p } } \right\rangle } \right\}$$

where $m_i = \left\langle {d_i ,b_i ,e_i } \right\rangle$ represents a medical-named entity mention that appears in the document $d_i$, $b_i$ and $e_i$ indicate the beginning and ending positions of $m_{i }$ in $d_i$ respectively, $c_{m_i } \in C$ indicates the assigned pre-defined category for $m_{i }$. The named entity mentions do not overlap, that is, $e_i < b_{i + 1}$. The predefined categories* C* about medical named entity in this study, as defined in [1], which are defined as follows:

1. **Diseases & diagnoses (DISEASES):** Medically defined diseases and the judgments made by doctors in clinical practice regarding etiology, pathophysiology, classification, and staging.
2. **Imaging examinations (EXAM):** Includes X-ray, CT scan, MRI, PET-CT, etc. It does not include diagnostic procedures such as gastroscopy and colonoscopy to avoid excessive conflict with surgical procedures.
3. **Lab tests (TEST):** Physical or chemical evaluations performed in the laboratory, specifically referring to clinical lab analyses undertaken by the laboratory division. It does not include immunohistochemistry and other broad laboratory tests.
4. **Surgeries (TREAT):** Surgical operations conducted by medical professionals on particular regions of the patient's body, including excision, suturing, and other treatments. It is the main treatment method in surgery.
5. **Medications (DRUG):** Specific chemical compounds utilized for curing illnesses.
6. **Anatomical sites (BODY):** Pertains to the anatomical places in the human body where diseases, symptoms, and signs manifest.

This research focuses on design models that improve the accuracy of recognizing the above six types of medical-named entities.

### Method Descriptions

**Fig. 1:**
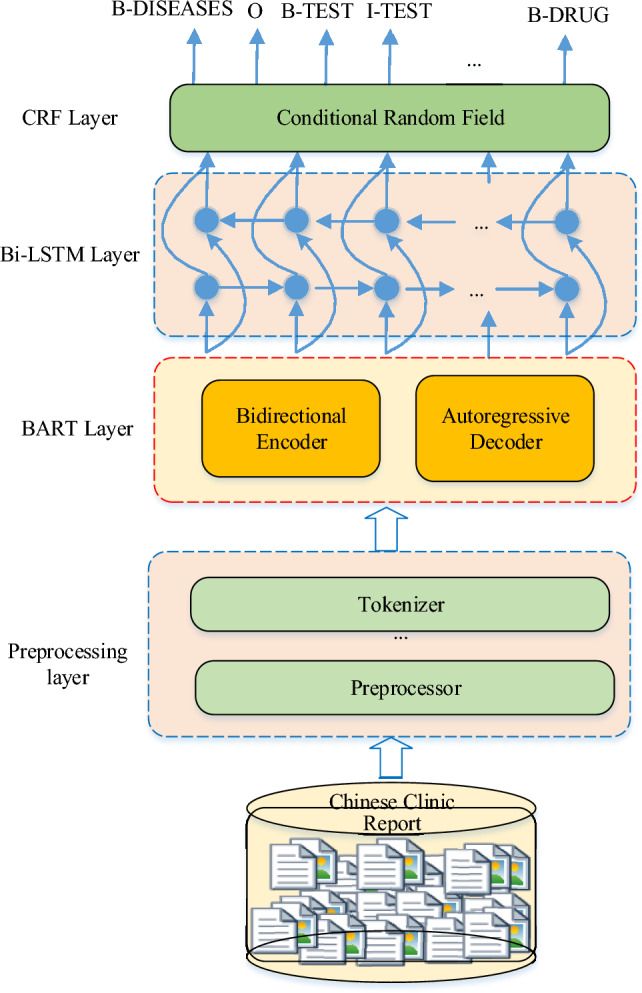
Multi-model fusion structure block diagram of MF-MNER

For the above task, we have designed a multi-model fusion method for Chinese clinical electronic MNER. The approach we put forward is composed of four layers: a preprocessing layer, a BART layer, a Bi-LSTM layer, and a CRF layer, as shown in Fig. 1.

First, the raw Chinese electronic medical record dataset is preprocessed using the preprocessing layer. This includes sentence segmentation, word segmentation, named entity annotation, and label sequence construction. The training set and validation set are obtained from the original data by random partitioning. Next, the preprocessed training data is fed into the next BART layer to convert the input sentences into fixed-length vector representations. The output of the MF-MNER model is obtained through a Bi-LSTM layer combined with a CRF layer for named entity labeling. This process generates the predicted segmentation results. The critical methods utilized in this research are detailed in the ensuing subsections.

#### The Preprocessing Layer

The initial stage of MF-MNER is the preprocessing layer, which is responsible for denoising, sentence segmentation, and word segmentation of the electronic medical records, as well as designing a labeling scheme to generate a standardized Chinese named entity recognition (CNER) dataset.

The most obvious marker for Chinese electronic medical record sentence recognition is the "period", so we first use the "period" as a delimiter to sequentially perform sequence sentence segmentation and corresponding label extraction from the original electronic medical record data. Then, we extract each character from the sentence and construct a list of characters and their corresponding labels to obtain the labeled dataset after "word segmentation" of the electronic medical records. We then use the BIO labeling strategy (Begin, Inside, Outside) to map the given labels to each character for character-level labeling to improve the accuracy of model predictions. Ultimately, we utilize the Hugging Face tokenizer to process this data and convert the electronic medical record text into tensor data that can be processed by the subsequent BART layer to match its processing requirements.

#### BART Layer

The second layer of MF-MNER is the BART layer, which is based on the BART model developed by Facebook [30]. It consists of a bidirectional encoder and an autoregressive decoder. BART samples random tokens and replaces them with masks. The decoder then uses the output of the encoder and the previously uncorrupted tokens to reconstruct the original document. This approach greatly improves the natural language processing capabilities. Some Chinese natural language processing researchers have also developed related Chinese pretraining models. Although these models have been applied in multiple domains, they are trained on Chinese Wikipedia and a subset of WuDaoCorpus [31]. They are not suitable for professional medical field scenarios.

To address this, we need to fine-tune BART layer by employing a limited amount of annotated data. After preprocessing the clinical electronic medical record data, every word *x*_i_ in the phrase can be transformed from a one-hot vector to a compact, high-density word vector *x*_i_ ∈ *R*_d_, where *R*_d_ represents the d-dimensional real number field, and the *x*_i_ embedding degree is the *d*-dimensional. During the pretraining of the BART model, multiple noise transformations are applied to the sequences to rebuild the original sequences from the distorted ones, leveraging the bidirectional self-learning ability of BART. This improves the robustness of the model and enables better prediction of named entity labels.

#### Bi-LSTM Layer

The Bi-LSTM layer is added after the BART layer to further boost the MF-MNER model's capacity to grasp and model the contextual data in the electronic medical record sequences. The Bi-LSTM [32] comprises two LSTM [33] networks, one handling the input sequence in the forward manner and the other in the reverse manner. At each time step, every LSTM unit in the Bi-LSTM layer has access to both the preceding and succeeding context information of the electronic medical records. This bidirectional reading capability allows the Bi-LSTM layer to better grasp the associations and relationships between the words in the sequences of electronic medical records. The method can capture not only the local context of every word but also the global context of the entire sequence by incorporating the Bi-LSTM layer. This enables the model to extract more meaningful features from the electronic medical record data, which can improve the accuracy and performance of the downstream tasks.

For every sentence, the word embedding sequence *x* = (*x*_1_, *x*_2_, …, *x*_m_) is fed into the Bi-LSTM at each step. The output sequence of hidden states from the forward LSTM $(\overrightarrow {h_1 } ,\overrightarrow {h_2 } ,...,\overrightarrow {h_m } )$ and the corresponding output sequence from the backward LSTM $(\overleftarrow {h_1 } ,\overleftarrow {h_2 } ,...,\overleftarrow {h_m } )$ are merged based on the position* h*_t_ = [$\overrightarrow {h_t }$; $\overleftarrow {h_t }$] ∈ *R*^n^ to produce the full sequence of hidden states: (*h*_1_, *h*_2_, …, *h*_m_) ∈ R^m×n^.

The role of the subsequent linear layer is to transition the hidden state vector from *n*-dimension to *k*-dimension, where *k* corresponds to the number of labels outlined in the tagging scheme. Consequently, the sentence attributes of electronic medical record data are extracted and represented as a matrix P = (*p*_1_, *p*_2_, …, *p*_n_) ∈ R^m×k^.

#### CRF Layer

The CRF layer on top of the BART-BiLSTM model can effectively use the transition matrix to capture the dependencies between labels in a sequence, which can reduce error propagation in the final predictions. According to Ref. [34], the CRF layer's parameters are represented by a matrix A ∈ R^(k+2)×(k+2)^. Each entry *A*_ij_ in the matrix denotes the score of the transition from the *i*th label to the *j*th label. Reflect on a series of labels *y* = (*y*_1_, y_2_, …, *y*_m_), the score of the sequence of labels is determined using Eq. (3):

3 $$score\left( {x,y} \right) = \mathop \sum \limits_{i = 1}^m P_{i,y_i } + \mathop \sum \limits_{j = 1}^{m + 1} A_{y_{j - 1} ,y_j }$$

The sequence's total score is calculated by adding up individual word scores. Each word in the sentence contributes to this score. The output matrix *P* from the Bi-LSTM layer plays a role in this calculation. Also, the transition matrix A from the CRF layer has an impact on the score.

The probability $P_{i,y_i }$ can be obtained by the Eq. (4) for a training sample (*x*, *y*^x^), during model training [34].

4 $$\begin{aligned} & \log P\left( {y^x \mid x} \right) = {\text{score}}\left( {x,y^x } \right) \\ & \quad - {\text{log}} \sum \limits_{y^{\prime} } \exp \left( {{\text{score}}\left( {x,y^{\prime} } \right)} \right) \\ \end{aligned}$$

The training process of the model involves maximizing the log-likelihood function. Additionally, the model uses the Viterbi algorithm, which utilizes dynamic programming, to determine the optimal path during prediction by Eq. (5).

5 $$y^{*} = {\mathop {{\text{arg}}\;{\text{max}}}\limits_{y^{\prime} }} \;{\text{score}}\left( {x,y^{\prime} } \right)$$

## Implementation Process

### Experimental Software and Hardware Environment

The experimental training for this study was conducted on an i9-11950H CPU and a NVIDIA GeForce RTX 3080 Laptop GPU (16G) setup. The training framework utilized was Python 3.7 and TensorFlow 1.14.0. The proposed approach was implemented using Python 3.7, the version of PyTorch was 1.13.1, and the version of PyTorch with CUDA support was 11.7, on a 64-bit virtual machine.

### Data Sources and Preprocessing

The experimental data utilized herein consists of two main parts: the first is the CCKS2019 dataset, and the other is an actual clinical dataset. The CCKS 2019 dataset was downloaded from the official website of WuDaoCloud (Beijing) Technology Co., Ltd (http://openkg.cn/dataset/yidu-s4k). The original CCKS 2019 dataset comprises 1,379 Chinese electronic medical record entries, which consist of 1,000 annotated records and 379 non-annotated records. The 1,000 records are primarily utilized for model training and validation, while the 379 non-annotated entries are chiefly employed for baseline data to assess model performance. The definitions of the six named entity types within this dataset are as delineated in Sect. 2.1. The actual data set comes from the collaborating hospital studied in this project, including 100 Chinese clinical electronic medical records, and is mainly used for further practical application testing of the designed method.

The raw electronic medical record data was preprocessed. The first step was to segment the text into sentences. The delimiter used for this segmentation was the Chinese "。" (full stop). After the initial processing, further refinement is required. Specifically, five steps are designed: (1) If the number sequence contains Chinese characters, split it; (2) If the special character is preceded by a newline character, we skip this operation and do not split it; (3) The combination of letters and numbers before and after cannot be cut; (4) If there are letters before and after, it should not be divided and retained at this time; (5) If there are numbers before and after a special symbol, it should not be separated and retained at this time. When performing preprocessing work, we need to constantly observe the original text output and then perform in-depth text preprocessing operations, especially Chinese data. Therefore, preprocessing is a very complex and important step, which determines the quality of subsequent experiments.

**Fig. 2:**
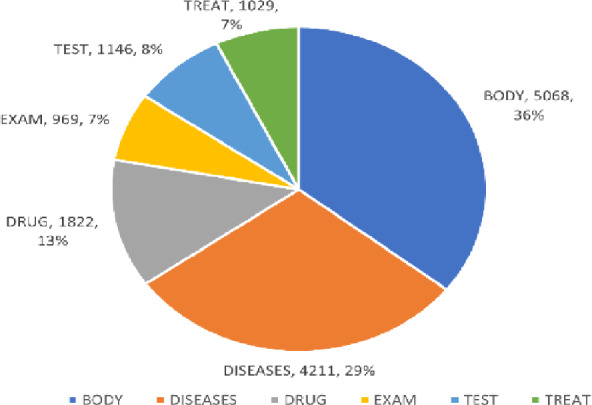
The Statistics number and the distribution of medical entities in the CCKS 2019 original data

Then, for each sentence, we extracted the corresponding labels and performed sequence sentence segmentation. Each sentence, consisting of *m* Chinese characters, was depicted as *x* = (*x*_1_, *x*_2_, …,* x*_i_, …, *x*_m_), and *x*_i_ was portrayed as the index of the* i*th Chinese character in the constructed vocabulary list. We further extracted individual characters from the sentences, constructing a list of characters and their corresponding labels. This process resulted in a labeled dataset of electronic medical records after character-level segmentation. The statistics, which are presented, are of the six named entity types (*DISEASES*, *EXAM*, *TEST*, *TREAT*, *DRUG*, *BODY*), and are derived from the original Chinese clinical electronic medical records dataset. Figure 2 provides a visual representation of these statistics.

According to the BIO (Begin, Inside, Outside) labeling strategy, the labels provided in the dataset were mapped to each character to perform character-level tagging, improving the accuracy of the model predictions. For example, if "X" represents a certain named entity category, the beginning is marked as "B-X", the middle and end characters are marked as "I-X", and other characters that are not part of a named entity word are marked as "O". In this study, there are a total of 13 labels corresponding to different entities: *B-DISEASES, B-EXAM*, *B-TEST*, *B-TREAT*, *B-DRUG*, *B-BODY I-DISEASES*, *I-EXAM*, *I-TEST*, *I-TREAT*, *I-DRUG*, *I-BODY*, *O*. There are only the first 12 labels by analyzed in this study as they are closely related to medical entities.

We have designated 256 as the maximum length for the sequence, and the Padding function is used to pad all sequences in a batch based on the maximum sequence length in that batch. Subsequently, the preprocessed 1000 electronic medical records are randomly divided into training and validation sets, adhering to an 8:2 proportion. The model vocabulary is tokenized using a Tokenizer. With these steps, the data preprocessing is completed.

### Settings for Each Layer of MF-MNER

After the above preprocessing, we use BART-based pre-training. First, Various noises are utilized to corrupt the original text, which is subsequently reconstructed via the seq2seq model, ultimately enhancing the preprocessed corpus quality. The Bart layer is designed based on "fnlp/bart-base-chinese" [31]. The vocabulary size is 51,271, which is a larger vocabulary built based on the training data.

The activation function of each layer's neuron uses the GeLUs function, and the initialization parameters obey the *N* (0, 0.02) distribution. The maximum sequence length and the maximum position embeddings are designated as 1024. The batch size is adjusted to 2048, and a 2e−5 learning rate and 0.1 warm-up ratio are used. The model architecture is based on a 12-layer stacked bidirectional Transformer with a 768-unit hidden layer and 12 attention heads. The BART model consists of a shared embedding layer and 6 Transformer encoder/decoder layers. The shared embedding layer maps the word indices in the input data sequence to word vector representations. The input dimensions of the embedding layer are the vocabulary size (51,271) and the word vector dimension (768). The padding_idx parameter is used to map the padding word indices to zero vectors. The 6 Transformer encoder layers are used to encode the input sequence. Each encoder layer is composed of a self-attention mechanism along with a pair of fully connected layers. The self-attention mechanism calculates the contextual vectors at every spot in the input sequence of the electronic medical record data. The fully connected layers perform non-linear transformations on the contextual vectors. The decoder part also consists of 6 Transformer decoder layers, which are used to generate the output sequence. Each decoder layer comprises a self-attention mechanism, an encoder-decoder attention mechanism, and a pair of fully connected layers. The self-attention mechanism calculates the contextual vectors of the electronic medical record data at each position in the decoder input sequence. The encoder-decoder attention mechanism determines the alignment between the sequence input to the decoder and the sequence output from the encoder, and the fully connected layers perform non-linear transformations on the contextual vectors to enhance the model's resilience.

The BART layer is followed by the Bi-LSTM layer, which has an input size of 768 and a hidden size of 128. It processes the input sequence of electronic medical record data, capturing the contextual information in the medical record data sequence.

The data processed by the Bi-LSTM layer is then fed into a CRF layer. The CRF layer utilizes the features extracted by the Bi-LSTM to perform named entity classification for each position, outputting the probability distribution of the labeled sequences. Based on the probability distribution, the named entity label corresponding to each position, denoted as y1, is determined based on the highest probability *p*i for each position *x*.

### Model Training, Hyperparameter Optimization

The AdamW optimize [35] is used in this paper to update the parameters. The pseudocode for the optimization process is as follows:
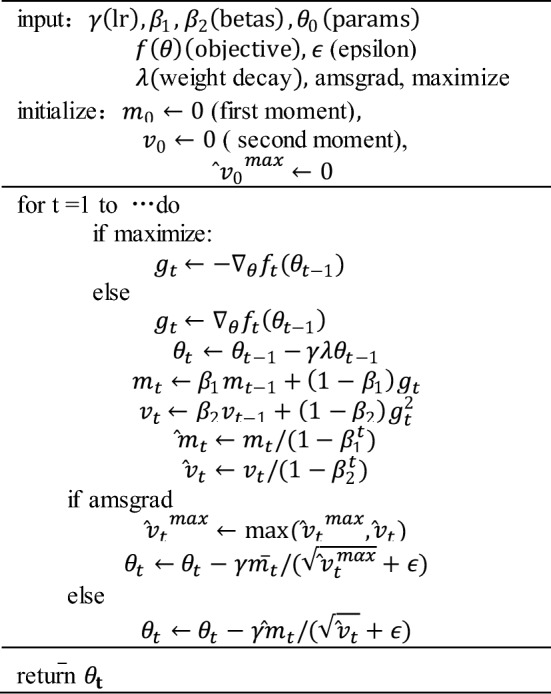


In the above pseudocode, *learning_rate* is the initial learning rate, *weight_decay* is the weight decay coefficient for L2 regularization. The optimizer initializes the first and second moment estimates *m* and *v* to zero. In each training step, the learning rate is updated by step *t* and the warmup steps. The optimizer is then zeroed out, and the loss is calculated for the current batch. The gradients are then calculated and backpropagated through the network. Finally, the optimizer updates the parameters with the calculated gradients and the weight decay coefficient.

**Fig. 3:**
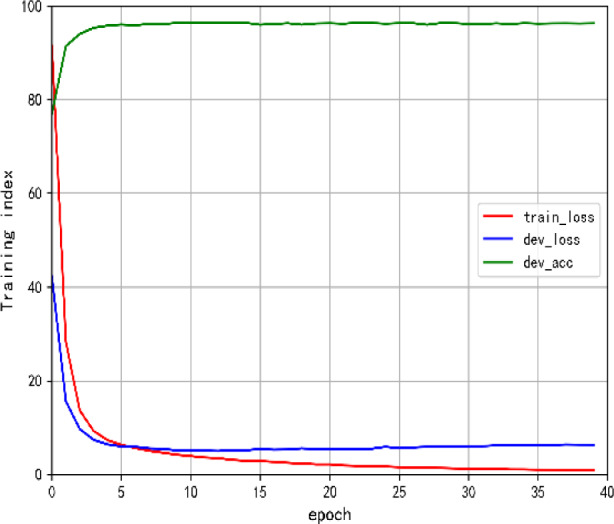
Performance curve of the training process (AdamW)

The key parameter values of our models are listed in Table 1. The training curves of various training metrics during the process of model training are depicted in Fig. 3.

**Table 1:** Parameter settings

Parameters	Value
Epoch	40
optimizer	AdamW
learning_rate (lr)	0.001
Batch size	64
Decay rate	0.8
Bi-LSTM_dim	768*128
Dropout	0.1
out_features (linear)	256
in_features (linear)	16
num_tags (CRF)	16

Looking at Fig. 3, it's evident that the model has achieved optimal results after about 40 batches of training, which greatly shortened the model training time and improved the model training efficiency.

## Performance Evaluation and Result Analysis

### Evaluation Metrics

This article involves six types of named entity recognition, including diseases & diagnosis, laboratory tests, imaging examinations, surgeries, drugs, and anatomical parts. For each category, it is a two-classification problem. Commonly used evaluation metrics include Recall rate(R), *Precision* rate (P), and F1 score, the calculation formulas of these metrics are as Eqs. (6–8) [36]:

6 $$R = \frac{TP}{{TP + FN}}$$

7 $$P = \frac{TP}{{TP + FP}}$$

8 $$F_1 = \frac{2PR}{{P + R}}$$

where *TP* is True Positives, refer to the medical entities correctly identified in the clinical electronic medical record text. *FP*, also known as False Positives, are instances where words, that are not entities, are incorrectly identified as such in the text. *FN* stands for False Negatives, which is when medical named entity words in the text are not recognized as entities.

To comprehensively evaluate the performance of our MF-MNER method for recognizing these 6 types of entities (12 labels) in clinical electronic medical records, we employed three evaluation mechanisms: micro-average, macro-average, and weighted-average [37]. The detailed calculation methods of these three evaluation mechanisms are as follows:

1. The micro-average (micro-avg) are obtained based on the statistical establishment of a global confusion matrix for each sample (regardless of category) in the clinical electronic medical record data set. Here we mainly use micro-avg to evaluate our method's overall performance on 6 types of medical entities. The calculation method of $$micro_\_P ({\text{Precision rate}})$$,$$micro_\_R$$ $$({\text{Recall rate}})$$ and $$micro_\_F1$$ (F1-score$$)$$ are defined as Eqs. (9–11):
  9 $$micro_\_P = \frac{{\sum_{i = 1}^n TP_i }}{{\sum_{i = 1}^n TP_i + \sum_{i = 1}^n FP_i }}$$
  10 $$micro_\_R = \frac{{\sum_{i = 1}^n TP_i }}{{\sum_{i = 1}^n TP_i + \sum_{i = 1}^n FN_i }}$$
  11 $$micro_\_F1 = \frac{2 \times micro_\_P \times micro_\_R}{{micro_\_P + micro_\_R}}$$
2. The macro averaging (macro-avg) involves first calculating the metrics for each category separately, and then uses the average of these category metrics as the final evaluation result. Here we use macro-avg to evaluate our method's performance when the average attention is paid to 6 types of entities. The calculation methods of $$macro_\_P$$ $$({\text{Precision rate}})$$, $$macro_\_R$$ $$({\text{Recall rate}})$$ and $$macro_\_F1$$ (F1-score$$)$$ are defined as Eqs. (12–14):
  12 $$macro_\_P = \frac{1}{n}\mathop \sum \limits_{i = 1}^n P$$
  13 $$macro_\_P = \frac{1}{n}\mathop \sum \limits_{i = 1}^n R_i$$
  14 $$macro_\_F1 = \frac{2 \times macro_\_P \times macro_\_R}{{macro_\_P + macro_\_R}}$$
3. The weighted average (weighted -avg) weights the metric based on the number of samples in each category to account for differences between categories and sample imbalance. The calculation method of $$weighted\_P$$ $$({\text{Precision rate}})$$, $$weighted\_R{ }({\text{Recall rate }})$$ and $$weighted\_F1$$ (F1-score$$)$$ are shown in Eqs. (15–17):
  15 $$weighted\_P = \frac{{\sum_{i = 1}^n w_i \times TP_i }}{{\sum_{i = 1}^n w_i \times TP_i + \sum_{i = 1}^n w_i \times FP_i }}$$
  16 $$weighted\_R = \frac{{\sum_{i = 1}^n w_i \times TP_i }}{{\sum_{i = 1}^n w_i \times TP_i + \sum_{i = 1}^n w_i \times FN_i }}$$
  17 $$weighted\_F1 = \frac{2 \times weighted\_P \times weighted\_R}{{weighted\_P + weighted\_R}}$$
  18 $$w_i = \frac{{num\_{\text{i}}}}{{num\_{\text{all}}}}$$

where *w*_i_ is the weight. *num_i* is the number of samples in each category. *num_all* is the total number of samples in all categories.

### Performance Analysis of the MF-MNER Model

**Fig. 4:**
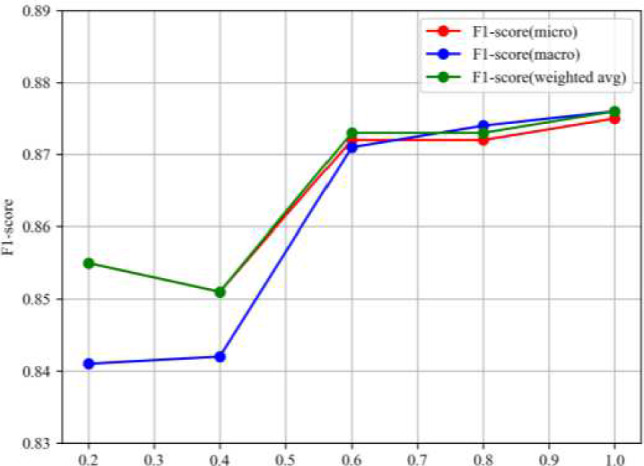
Comparing *F*1*-scores* of three kinds under various training set sizes (CCKS2019 dataset). The horizontal axis signifies the ratio of the training set to the total training data. The ratios of the training data and the total number obtained in this study are 0.2, 0.4, 0.6, 0.8, and 1 respectively

In this subsection, our proposed model is validated by testing on CCKS 2019 datasets using *Precision*, *Recall*, and *F*1-*score*. There are 6 types of entities, 12 types of prediction Results, and comprehensive evaluation metrics are listed in Table 2 and shown Fig. 4.

**Table 2:** The prediction results of 12 types on CCKS2019 dataset with MF-MNER(our method)

Entity name	*Precision*	*Recall*	*F*1-*score*
*B-BODY*	0.886	0.916	0.900
*B-DISEASES*	0.869	0.860	0.864
*B-DRUG*	**0.939**	**0.925**	**0.932**
*B-EXAM*	0.858	0.843	0.851
*B-TEST*	0.845	0.781	0.812
*B-TREAT*	0.921	0.858	0.888
*I-BODY*	0.763	0.869	0.812
*I-DISEASES*	0.888	0.857	0.872
*I-DRUG*	**0.964**	**0.923**	**0.943**
*I-EXAM*	0.849	0.880	0.864
*I-TEST*	0.879	0.761	0.816
*I-TREAT*	**0.980**	**0.928**	**0.953**

Bold represents rows where all indicators exceed 0.9, indicating higher recognition accuracy for these entities relative to the rest

From Table 2, its shows that the *Precision*, *Recall*, *F*1*-score* of *B-DRUG* reach 0.939, 0.925, 0.932, and the *Precision*, *Recall* and *F*1-*score* of *I-DRUG* reach 0.964, 0.923, 0.943. The *Precision*, *Recall* and *F*1-*score* of *I-TREAT* are 0.921, 0.858, and 0.888. The MF-MNER has achieved a value of more than 0.9 for the three metrics *B-DRUG*, *I-DRUG*, and *I-TREAT*, which is higher than the other 9 tags. The reason for this effect may be that the public corpus has more information about these three types of named entity tags.

To assess the comprehensive effectiveness of MF-MNER, the three evaluation metrics of *micro-average* (*micro avg*), *macro-average* (*macro avg*) and *weighted-average* (*weighted avg*) are calculated respectively using Eqs. (11–17). The results are shown in Table 3.

**Table 3:** Comparison of performance indicators of *Precision*, *Recall,* and *F*1-*sore* MF-MNE on CCKS2019 dataset under three evaluation mechanisms

Name	*Precision*	*Recall*	*F*1*-score*
*micro avg*	0.880	0.871	0.875
*macro avg*	0.887	0.867	0.876
*weighted avg*	0.883	0.871	0.876

From Table 3, it is find that all metrics reached a relatively close level. The score indicates that the comprehensive performance of MF-MNER is good whether it is from the perspective of focusing on entities, focusing on entities on average, or focusing on entities weighted according to named entity distribution.

### Comparison of MF-MNER and Unoptimized BERT-BiLSTM-CRF Model

In order to evaluate the effectiveness of the design MF-MNER method, the existed model BERT-BiLSTM-CRF [29] is selected as the baseline comparison, because this document uses the same data set as we do, and because this document does not explain the calculation method of the listed writing metrics, we list all the metrics in Table 2 for comparison. The comparison results are shown in Table 4.

**Table 4:** Comparison of *Precision*, *Recall* and *F*1-*sore* between MF-MNER and BERT-BiLSTM-CRF on CCKS2019 dataset

Name	*Precision*	*Recall*	*F*1-*score*
BERT-BiLSTM-CRF [29]	0.738	0.753	0.745
MF-MNER(Micro avg)	0.880	0.871	0.875
MF-MNER (Macro avg)	0.887	0.867	0.876
MF-MNER (Weighted avg)	0.883	0.871	0.876

From Table 4, the three metrics of *Precision*, *Recall* and *F*1-*score* of the model we designed in the case of micro avg evaluation are better than the existing BERT-BiLSTM-CRF method [29] improved by 19.24%, 20.19% and 19.65% respectively. The three metrics of accuracy, recall and *F*1 score in the case of macro avg rating are respectively improved by 15.67%, 15.14% and 15.67% in comparison to the presented BERT-BiLSTM-CRF model [29]. In the case of weighted avg evaluation, the three metrics of accuracy and recall FI score are 17.45%, 17.58% and 17.58% higher than the existing BERT-BiLSTM-CRF model [29] respectively. These results illustrates that the overall performance of MF-MNER is superior to existed method.

### Performance of Our Optimized BERT-BiLSTM-CRF Model

To further study, we use AdamW to improve the Bert-BiLSTM-CRF [29] model performance. Under the same experimental environment conditions as our model, the same batch training settings are adopted. The obtained prediction result metrics of the 12 named entity tags are shown in Table 5.

**Table 5:** Recognition results of *Precision*, *Recall* and *F*1-*sore* about 12 named entity tags by BERT-BiLSTM-CRF model on CCKS2019 dataset after our optimization

Name	*Precision*	*Recall*	*F1-score*
*B-BODY*	0.880	0.910	0.895
*B-DISEASES*	0.857	0.851	0.854
***B-DRUG***	**0.942**	**0.879**	**0.909**
*B-EXAM*	0.848	0.826	0.837
*B-TEST*	0.849	0.689	0.761
*B-TREAT*	0.878	0.802	0.839
*I-BODY*	0.753	0.860	0.803
*I-DISEASES*	0.888	0.830	0.858
***I-DRUG***	**0.974**	**0.888**	**0.929**
*I-EXAM*	0.837	0.872	0.854
*I-TEST*	0.881	0.691	0.774
***I-TREAT***	**0.975**	**0.903**	**0.937**

Bold represents rows where all indicators exceed 0.9, indicating higher recognition accuracy for these entities relative to the rest

To analyze the optimized model overall performance, we also calculated the three metrics of *micro avg* (macro-average), *macro avg* (micro-average) and *weighted avg* (weighted-average) respectively using Eqs. (11–17), Table 6 shows the results.

**Table 6:** Comparison of performance indicators of *Precision*, *Recall* and *F*1-*sore* our optimized Bert-BiLSTM-CRF’s on CCKS2019 dataset under three evaluation mechanisms

Evaluation mechanisms	*Precision*	*Recall*	*F*1*-score*
*micro avg*	0.876	0.845	0.860
*macro avg*	0.880	0.833	0.854
*weighted avg*	0.880	0.845	0.860

From Table 6, the optimized metrics have far exceeded the metrics of the baseline model [29], indicating that AdamW’s optimization method is very suitable for this type of medical clinical electronic medical record data, whether it is from the overall focus on the named entity, from the average focus from the perspective of entities, or from the perspective of focusing on entities based on weighted named entity distribution, the overall model has been greatly improved.

### Performance Comparison of Some Existed Models on CCKS2019 Dataset

To demonstrate the superiority of the MF-MNER method designed in this paper, we conducted comparative experiments on the same dataset CCKS2019, mainly comparing our designed MF-MNER, Ref. [29], our optimized Ref. [29] model, and the method in Ref. [28].To be fair, we used the weighted avg evaluation metric value in our designed model for comparison (Refs. [28, 29] did not specify which evaluation mechanism(*micro avg, macro avg and weighted avg*) was used). Table 7 displays the results of the comparison.

**Table 7:** Comparison of *Precision*, *Recall* and *F*1-*sore* of some existed models on CCKS2019 dataset under the weighted avg evaluation mechanism

Model	*Precision*	*Recall*	*F*1-*score*
Lattice-LSTM^a^-CRF^b^ [28]	0.851	0.833	0.842
Random-lattice-LSTM-CRF [28]	0.851	0.837	0.844
GloVe^c^ -lattice-LSTM-CRF [28]	0.853	0.839	0.846
ELMo^d^ -lattice-LSTM-CRF (ML^e^) [28]	0.822	0.841	0.832
**ELMo-lattice-LSTM-CRF** [28]	**0.847**	**0.854**	**0.850**
BERT-BiLSTM-RFC [29]	0.738	0.753	0.745
Our optimized [29]	0.880	0.845	0.860
**MF-MNER**	**0.883**	**0.871**	**0.876**

Bold represents rows where all indicators exceed 0.9, indicating higher recognition accuracy for these entities relative to the rest

^a^LSTM: long short-term memory

^b^CRF: conditional random field

^c^GloVe: Global Vectors for Word Representation

^d^ELMo: Embeddings from Language Models

^e^ML: Many languages

Under the condition of datasets of equal size, the MF-MNER method proposed in this study has improved the *Precision*, *recall*, and *F*1-*score* by 19.24%, 12.22%, and 15.44%, respectively, compared to our optimized Ref. [29] method. In contrast to the baseline model, the three metrics of our proposed method have seen enhancements of 19.65%, 15.67%, and 17.58% respectively. Compared with the best method obtained in Ref. [28], the MF-MNER method proposed in this study improved the *Precision*, *Rcall*, and *F*1 *score* by 4.25%, 2.00%, and 3.05%, respectively.

### Comparative Analysis of MF-MNER Model on the CCKS2019 Dataset with Varying Training Set Sizes

To further verify the performance of MF-MNER in small data, we conducted comparative experiments, randomly extracting 20%, 40%, 60%, and 80% of the total training data without replacement and all data for comparison. The comparison of *F*1 *score* results under different data sets is shown in Fig. 4.

Figure 4 shows that when the training data is multiplied by 0.6 train set ratio of the total training data, our MF-MNER has achieved good performance, and the *F*1 score is close to the optimal level. This indicates that our MF-MNER is better adapted for named entity recognition in medical electronic records in small datasets.

### Performance Comparison of MF-MNER Model and Our Optimized Bert-BiLSTM-CRF on the Real Data Set

To further validate the performance of the MF-MNER method designed in this study, we get a real data set, which contains 100 Chinese clinical electronic medical records from a tertiary hospital affiliated with Xinxiang Medical University (the First Affiliated Hospital of Xinxiang Medical University), with which we collaborate. After the same preprocessing, the MF-MNER model was evaluated under three assessment scenarios: micro average, macro average, and weighted average. Comparative experiments were conducted against the optimized method from Ref. [29]. The results are presented respectively in Table 8.

**Table 8:** Performance comparison of MF-MNER and our optimized Bert-BiLSTM-CRF on a real Chinese clinical electronic medical records dataset under the three kinds of evaluation mechanism

Evaluation mechanism	Methods	*Precision*	*Recall*	*F*1-*score*
micro-avg	Our optimized [29]	0.627	0.800	0.703
micro-avg	MF-MNER	0.638	0.825	0.719
macro-avg	Our optimized [29]	0.669	0.769	0.710
macro-avg	MF-MNER	0.685	0.800	0.733
weighted avg	Our optimized [29]	0.638	0.800	0.705
weighted avg	MF-MNER	0.647	0.825	0.722

From Table 8, it can be observed that our designed MF-MNER method shows an improvement over our optimized version of the method described in Ref. [29] across all three evaluation criteria: micro avg, macro avg, and weighted avg. In terms of *Precision*, *Recall*, and *F*1-*score*, the enhancements are evident. Specifically, there is an increase of 1.75%, 3.12%, and 2.28% in the micro avg category, respectively. In the macro avg category, the increments are 2.39%, 4.03%, and 3.24%, respectively. Lastly, in the weighted avg category, the improvements are 1.41%, 3.12%, and 2.41%, respectively.

## Conclusion

In this study, a Chinese electronic medical record named entity identification approach, named MF-MNER, was developed inspired by the principle of model fusion. The main contributions of this paper can be summarized as follows:

1. Leveraging the encoder-decoder architecture of BART for its robust capabilities in understanding and articulating complex contextual information, as well as handling ambiguous entity boundaries during pre-training, to dynamically integrate context information from clinical electronic medical records, thereby enhancing fine-tuning efficiency and accomplishing the task of clinical electronic medical record entity recognition with minimal training data.
2. Design a novel MF-MNER model for entity recognition in Chinese medical electronic medical records. Initially, the Chinese electronic medical record data undergo preprocessing and encoding. Subsequently, the contextually informed and Bidirectionally Auto-Regressive Transformer (BART) pre-trained model is selected. This model is then refined using the AdamW optimization algorithm. The output from the BART layer is subsequently fed into the next layer, a Bidirectional Long Short-Term Memory (Bi-LSTM). The Bi-LSTM exploits its capability to process sequences in both forward and reverse directions, thereby integrating the sequence's "past" and "future" information, which is then "concatenated" to serve as the input for the CRF layer. Ultimately, the CRF layer employs its parameterized "transition matrix" and the Viterbi algorithm for decoding, to identify the most fitting annotation sequence for six types (Diseases and diagnoses (DISEASES), Imaging Examinations (EXAM), TEST (Lab tests), Surgeries (TREAT), Medications (DRUG), Anatomical sites (BODY)) of entities in Chinese clinical electronic medical records.
3. In the standard public CCKS2019 dataset, our proposed MF-MNER outperforms the existing literature in all three evaluation scenarios: micro-average, macro-average, and weighted-average. Compared to the existing BERT-BiLSTM-CRF model, our method has achieved significant improvements of 19.64% in *Precision*, 15.67% in *Recall*, and 17.58% in *F*1-*score*. When tested on a real-world Chinese clinical electronic medical record dataset from hospitals, our MF-MNER demonstrated a noticeable enhancement across all three evaluation scenarios in terms of *Precision*, *Recall*, and F1-score metrics, relative to our optimized BERT-BiLSTM-CRF approach. This further substantiates the effectiveness of our designed MF-MNER.

This study also has certain shortcomings and limitations. Specifically, the assessment experiments were only conducted on the CCKS2019 dataset and a collection of 100 authentic clinical data points for actual MF-MNER testing. Although there was a significant enhancement in model performance on the standard CCKS2019 dataset, the entity recognition performance on real medical data requires further improvement. Our subsequent efforts will be directed towards gathering a more extensive compilation of datasets from various hospitals, investigating methods to improve the model's learning and generalization capabilities, and addressing the issue of clinical information isolation between different domestic hospitals.

## Data Availability

The data in this study has been desensitized and does not involve usability issues.

## References

[1] Janett RS, Yeracaris PP (2020) Electronic Medical Records in the American Health System: challenges and lessons learned. Ciencia Saude Coletiva 25(4):1293–1304. 10.1590/1413-81232020254.28922019 32267432 10.1590/1413-81232020254.28922019

[2] Cerchione R, Centobelli P, Riccio E et al (2023) Blockchain’s coming to hospital to digitalize healthcare services: Designing a distributed electronic health record ecosystem. Technovation 120:102480. 10.1016/j.technovation.2022.102480 10.1016/j.technovation.2022.102480

[3] Edara DC, Vanukuri LP, Sistla V et al (2023) Sentiment analysis and text categorization of cancer medical records with LSTM. J Ambient Intell Humaniz Comput 14(5):5309–5325. 10.1007/s12652-019-01399-8 10.1007/s12652-019-01399-8

[4] Sutton RT, Pincock D, Baumgart DC et al (2020) An overview of clinical decision support systems: benefits, risks, and strategies for success. NPJ Digit Med 3(1):17. 10.1038/s41746-020-0221-y 32047862 10.1038/s41746-020-0221-y PMC7005290

[5] Desai RJ, Wang SV, Vaduganathan M et al (2020) Comparison of machine learning methods with traditional models for use of administrative claims with electronic medical records to predict heart failure outcomes. JAMA Netw Open 3(1):e1918962. 10.1001/jamanetworkopen.2019.18962 31922560 10.1001/jamanetworkopen.2019.18962 PMC6991258

[6] Fawzy AM, Rivera-Caravaca JM, Underhill P et al (2023) Incident heart failure, arrhythmias and cardiovascular outcomes with sodium-glucose cotransporter 2 (SGLT2) inhibitor use in patients with diabetes: insights from a global federated electronic medical record database. Diabetes Obes Metab 25(2):602–610. 10.1111/dom.14854 36054168 10.1111/dom.14854 PMC10087187

[7] Huang L, Shea AL, Qian H (2019) Patient clustering improves efficiency of federated machine learning to predict mortality and hospital stay time using distributed electronic medical records. J Biomed Inform 99:103291. 10.1016/j.jbi.2019.103291 31560949 10.1016/j.jbi.2019.103291

[8] Zeng J, Gensheimer MF, Rubin DL et al (2022) Uncovering interpretable potential confounders in electronic medical records. Nat Commun 13(1):1014. 10.1038/s41467-022-28546-8 35197467 10.1038/s41467-022-28546-8 PMC8866497

[9] Bannour N, Wajsbürt P, Rance B et al (2022) Privacy-preserving mimic models for clinical named entity recognition in French. J Biomed Inform 130:104073. 10.1016/j.jbi.2022.104073 35427797 10.1016/j.jbi.2022.104073

[10] Bhatia P, Celikkaya B, Khalilia M et al (2019) Comprehend medical: a named entity recognition and relationship extraction web service. In: 2019 18th IEEE international conference on machine learning and applications (ICMLA), Boca Raton, FL, USA, pp 1844–1851. 10.1109/ICMLA.2019.00297

[11] Zhu P, Cheng D, Yang F et al (2022) Improving Chinese named entity recognition by large-scale syntactic dependency graph. IEEE/ACM Trans Audio Speech Lang Process 30:979–991. 10.1109/TASLP.2022.3153261 10.1109/TASLP.2022.3153261

[12] Ji B, Liu R, Li S et al (2019) A hybrid approach for named entity recognition in Chinese electronic medical record. BMC Med Inform Decis Mak 19(2):149–158. 10.1186/s12911-019-0767-2 30961597 10.1186/s12911-019-0767-2 PMC6454595

[13] Ravikumar J, Kumar PR (2021) Machine learning model for clinical named entity recognition. Int J Electr Comput Eng 11(2):1689–1696. 10.11591/ijece.v11i2.p

[14] Chen X, Shi S, Zhan S et al (2019) Named entity recognition of Chinese electronic medical records based on cascaded conditional random field. In: 2019 IEEE 4th International Conference on Big Data Analytics (ICBDA). IEEE, pp 364–368. 10.1109/ICBDA.2019.8713244

[15] Yan X, Xiong X, Cheng X et al (2021) HMM-BiMM: hidden Markov model-based word segmentation via improved Bi-directional Maximal Matching algorithm. Comput Electr Eng 94:107354. 10.1016/j.compeleceng.2021.107354 10.1016/j.compeleceng.2021.107354

[16] Govindarajan S, Mustafa MA, Kiyosov S et al (2023) An optimization based feature extraction and machine learning techniques for named entity identification. Optik 272:170348. 10.1016/j.ijleo.2022.170348 10.1016/j.ijleo.2022.170348

[17] Wan Q, Liu J, Wei L et al (2020) A self-attention based neural architecture for Chinese medical named entity recognition. Math Biosci Eng 17(4):3498–3511. 10.3934/mbe.2020197 32987540 10.3934/mbe.2020197

[18] Qiu J, Zhou Y, Wang Q et al (2019) Chinese clinical named entity recognition using residual dilated convolutional neural network with conditional random field. IEEE Trans Nanobiosci 18(3):306–315. 10.1109/TNB.2019.2908678 10.1109/TNB.2019.2908678 30946674

[19] An Y, Xia X, Chen X et al (2022) Chinese clinical named entity recognition via multi-head self-attention based BiLSTM-CRF. Artif Intell Med 127:102282. 10.1016/j.artmed.2022.102282 35430042 10.1016/j.artmed.2022.102282

[20] Yu X, Hu W, Lu S et al (2019) BioBERT based named entity recognition in electronic medical record. In: 2019 10th international conference on information technology in medicine and education (ITME). IEEE, pp 49–52. 10.1109/ITME.2019.00022

[21] Gao S, Kotevska O, Sorokine A et al (2021) A pre-training and self-training approach for biomedical named entity recognition. PLoS ONE 16(2):e0246310. 10.1371/journal.pone.0246310 33561139 10.1371/journal.pone.0246310 PMC7872256

[22] Liu N, Hu Q, Xu H et al (2021) Med-BERT: a pretraining framework for medical records named entity recognition. IEEE Trans Ind Inf 18(8):5600–5608. 10.1109/TII.2021.3131180 10.1109/TII.2021.3131180

[23] Zheng Y, Han Z, Cai Y et al (2022) An imConvNet-based deep learning model for Chinese medical named entity recognition. BMC Med Inform Decis Mak 22(1):303. 10.1186/s12911-022-02049-4 36411432 10.1186/s12911-022-02049-4 PMC9677659

[24] Wang Y, Sun Y, Ma Z et al (2020) Application of pre-training models in named entity recognition. In: 2020 12th international conference on intelligent human–machine systems and cybernetics (IHMSC). IEEE, pp 23–26. 10.1109/IHMSC49165.2020.00013

[25] Tang B, Wang X, Yan J et al (2019) Entity recognition in Chinese clinical text using attention-based CNN-LSTM-CRF. BMC Med Inform Decis Mak 19(3):74. 10.1186/s12911-019-0787-y 30943972 10.1186/s12911-019-0787-y PMC6448175

[26] Gong L, Zhang Z, Chen S (2020) Clinical named entity recognition from Chinese electronic medical records based on deep learning pretraining. J Healthc Eng 2020:1–8. 10.1155/2020/8829219 10.1155/2020/8829219 PMC7707942 33299537

[27] Li L, Zhao J, Hou L et al (2019) An attention-based deep learning model for clinical named entity recognition of Chinese electronic medical records. BMC Med Inform Decis Mak 19:1–11. 10.1186/s12911-019-0933-6 31801540 10.1186/s12911-019-0933-6 PMC6894110

[28] Li Y, Wang X, Hui L et al (2020) Chinese clinical named entity recognition in electronic medical records: development of a lattice long short-term memory model with contextualized character representations. JMIR Med Inform 8(9):e19848. 10.2196/19848 32885786 10.2196/19848 PMC7501578

[29] Dai Z, Wang X, Ni P et al (2019) Named entity recognition using BERT BiLSTM CRF for Chinese electronic health records. In: 2019 12th international congress on image and signal processing, biomedical engineering and informatics (cisp-bmei). IEEE, pp 1–5. 10.1109/CISP-BMEI48845.2019.8965823

[30] Lewis M, Liu Y, Goyal N et al (2019) Bart: denoising sequence-to-sequence pre-training for natural language generation, translation, and comprehension. 10.48550/arXiv.1910.13461

[31] Shao Y, Geng Z, Liu Y et al (2021) Cpt: a pre-trained unbalanced transformer for both chinese language understanding and generation. 10.48550/arXiv.2109.05729

[32] Pirani M, Thakkar P, Jivrani P et al (2022) A comparative analysis of ARIMA, GRU, LSTM and BiLSTM on financial time series forecasting. In: 2022 IEEE International Conference on Distributed Computing and Electrical Circuits and Electronics (ICDCECE). IEEE, pp 1–6. 10.1109/ICDCECE53908.2022.9793213

[33] Hochreiter S, Schmidhuber J (1997) Long short-term memory. Neural Comput 9(8):1735–1780. 10.1162/neco.1997.9.8.1735 9377276 10.1162/neco.1997.9.8.1735

[34] Sutton C, McCallum A (2012) An introduction to conditional random fields. Found Trends® Mach Learn 4(4):267–373. 10.1561/2200000013 10.1561/2200000013

[35] Gupta A, Ramanath R, Shi J et al (2021) Adam vs. sgd: closing the generalization gap on image classification. In: OPT2021: 13th Annual Workshop On Optimization For Machine Learning. https://www.opt-ml.org/papers/2021/paper53.pdf

[36] Yacouby R, Axman D (2020) Probabilistic extension of precision, recall, and f1 score for more thorough evaluation of classification models. In: Proceedings of the first workshop on evaluation and comparison of NLP systems, pp 79–91. 10.18653/v1/2020.eval4nlp-1.9

[37] Dev VA, Eden MR (2019) Gradient boosted decision trees for lithology classification. Comput Aided Chem Eng 47:113–118. 10.1016/B978-0-12-818597-1.50019-9 10.1016/B978-0-12-818597-1.50019-9

